# Research on Rice Fields Extraction by NDVI Difference Method Based on Sentinel Data

**DOI:** 10.3390/s23135876

**Published:** 2023-06-25

**Authors:** Jinglian Tian, Yongzhong Tian, Yan Cao, Wenhao Wan, Kangning Liu

**Affiliations:** 1 Chongqing Jinfo Mountain Karst Ecosystem National Observation and Research Station, School of Geographical Sciences, Southwest University, Chongqing 400715, China; tjl1012@email.swu.edu.cn (J.T.);; 2 Chongqing Engineering Research Center for Remote Sensing Big Data Application, School of Geographical Sciences, Southwest University, Chongqing 400715, China; 3 Daotian Science and Technology Limited Company, Chongqing 400700, China; 4 Chongqing Geomatics and Remote Sensing Center, Chongqing 400715, China

**Keywords:** extraction of rice fields, NDVI, NDWI, rice harvesting period, Chongqing

## Abstract

To meet the challenge of food security, it is necessary to obtain information about rice fields accurately, quickly and conveniently. In this study, based on the analysis of existing rice fields extraction methods and the characteristics of intra-annual variation of normalized difference vegetation index (NDVI) in the different types of ground features, the NDVI difference method is used to extract rice fields using Sentinel data based on the unique feature of rice fields having large differences in vegetation between the pre-harvest and post-harvest periods. Firstly, partial correlation analysis is used to study the influencing factors of the rice harvesting period, and a simulation model of the rice harvesting period is constructed by multiple regression analysis with data from 32 sample points. Sentinel data of the pre-harvest and post-harvest periods of rice fields are determined based on the selected rice harvesting period. The NDVI values of the rice fields are calculated for both the pre-harvest and post-harvest periods, and 33 samples of the rice fields are selected from the high-resolution image. The threshold value for rice field extraction is determined through statistical analysis of the NDVI difference in the sample area. This threshold was then utilized to extract the initial extent of rice fields. Secondly, to address the phenomenon of the “water edge effect” in the initial data, the water extraction method based on the normalized difference water index (NDWI) is used to remove the pixels of water edges. Finally, the extraction results are verified and analyzed for accuracy. The study results show that: (1) The rice harvesting period is significantly correlated with altitude and latitude, with coefficients of 0.978 and 0.922, respectively, and the simulation model of the harvesting period can effectively determine the best period of remote sensing images needed to extract rice fields; (2) The NDVI difference method based on sentinel data for rice fields extraction is excellent; (3) The mixed pixels have a large impact on the accuracy of rice fields extraction, due to the water edge effect. Combining NDWI can effectively reduce the water edge effect and significantly improve the accuracy of rice field extraction.

## 1. Introduction

With population growth, advancing urbanization, and global changes, society’s growing food demand has put enormous pressure on the food supply [1]. Rice is an important food crop, with nearly half of the world’s population relying on it as a staple food. Since the 21st century, the per unit area growth rate and area of potential expansion for global rice yields have become smaller [2,3,4], affecting global food security. Accurate, fast and easy access to rice field information is, therefore, essential for estimating rice yields, planning and addressing global food security challenges.

A large number of studies have used Landsat (land remote sensing satellite), MODIS (moderate resolution imaging spectroradiometer) and Sentinel-2 data for rice field data extraction [5,6,7,8]. Existing methods can be divided into two categories: integrated extraction and individual extraction. Integrated extraction uses land use data to distinguish the types of different ground features, such as GlobeLand30, ESRI Land Cover data and national land survey data, among others. Individual extraction uses machine learning combined with the characteristics of the rice fields, such as computer classification methods, extraction using the phenological characteristics of rice fields, and a combination of the two [9,10,11]. MODIS and Landsat data have been used to obtain 30 m spatial resolution NDVI data at an 8-day interval through the calculation of NDVI, the application of the Savitzky-Golay (S-G) filter, and the acquisition of rice phenology information [12]. In a separate study, MODIS and Landsat data were fused using the spatial–temporal adaptive reflectance fusion model (STARFM) to obtain rice phenology information from NDVI time series and Land Surface Temperature (LST) data from multi-temporal Landsat data. Finally, based on the multi-temporal Landsat data, the phenology data and the LST data, the rice planting area was extracted using the convolutional neural network (CNN) model. This method has the potential to extract rice field information based on medium-resolution images, but it does not consider the spatial pattern of the study areas. Higher spatial heterogeneity can lead to the appearance of mixed pixels and the distortion of land cover boundaries and contours [13]. For regions with scattered distribution of rice fields, undulating terrain and complex landscape types, rice data have been extracted by combining CNN models and decision tree (DT) with phenological indicators. However, the CNN structure and optimal parameters in this method need to be adjusted for the situation of the study area and the resolution of the remote sensing images, while the accuracy of phenological indicators depends on the weather conditions [14]. Few studies have used only specific phenological periods and a small number of indicators to study the distribution of rice.

In this study, the major purpose is to address the intricate procedures, extensive data requirements, and high technical demands that pertain to the extraction of rice fields. Based on an analysis of existing methods for extracting rice fields and the characteristics of intra-annual variation of NDVI in the different types of ground features, the article proposes a method for extracting rice fields using the NDVI difference, leveraging the unique feature of rice fields having large differences in vegetation between the pre-harvest and post-harvest periods.

## 2. Materials and Methodology

### 2.1. Study Area

**Figure 1:**
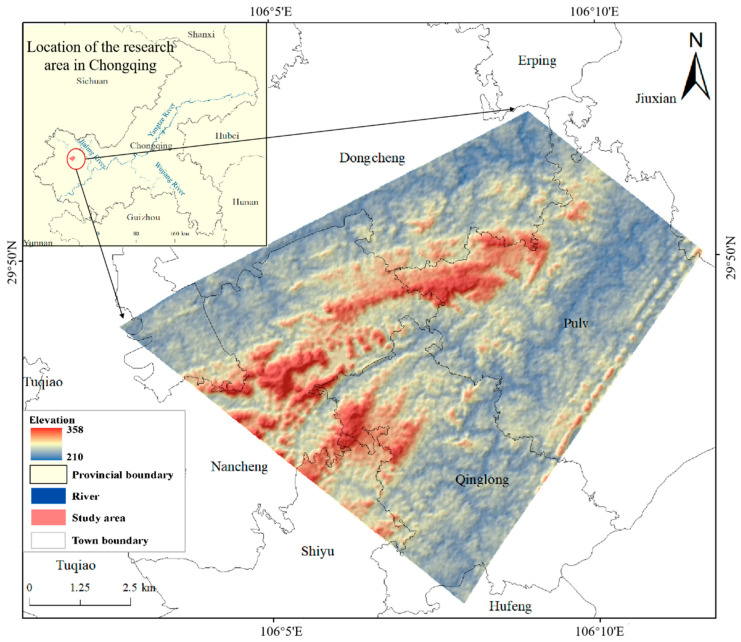
The location and topography of the study area.

The study area is located in the western part of Chongqing, in the transition zone from the Paralleled Ridge-Valley of East Sichuan to the Fangshan hills of central Sichuan (between 106°2′ to 106°12′ E and 29°45′ to 29°52′ N) (Figure 1). The total area is approximately 86.81 km^2^. The terrain is mainly hilly, higher in the center and lower in the valley, with the Xiao’an River on the eastern side and the Huaiyuan River on the western side. The elevation ranges from 210 m to 358 m, with average elevation of approximately 242 m and average slope of approximately 6°. The area has a subtropical, humid monsoon climate, with an early spring and hot summer, rainy autumn and warm winter. The average annual temperature is approximately 18 °C. Precipitation is concentrated from May to September, with August minimally impacted by the summer drought [15]. The main ground types are arable land, forest land, water bodies, urban industrial, and urban mining and construction land. The east and west sides of the study area are rural. Rice and corn are the main crops, with rice grown once a year, called one-season rice.

### 2.2. Data Source and Pre-Processing

The data sources of this study include Sentinel-2 data (European Space Agency); SuperView-1 satellite data (China Aerospace Science and Technology Corporation., Beijing, China); Digital elevation data (National Aeronautics and Space Administration., Washington, DC, USA); and rice harvesting period data. First, Sentinel-2 deploys two high-resolution multispectral optical satellites, namely Sentinel-2A and Sentinel-2B [16]. They are single satellites with revisit cycles of 10 days and two complementary 5-day cycles, respectively, carrying a Multispectral Imagery sensor for land monitoring [17,18,19]. The sensor offers 13 spectral wavebands: B2, B3, B4 and B8 which have a resolution of 10 m; B5, B6, B7, B8A, B11 and B12 have a resolution of 20 m; B1, B9 and B10 have a resolution of 60 m. Radiometric calibrations and atmospheric corrections are performed using the Sen2Cor plug-in [20]. At the same time, resampling and band fusion are essential.

Second, SuperView-1 satellite data were employed predominantly for verification in this study, which is a commercially developed remote sensing satellite manufactured by China. Its specifications include a panchromatic resolution of 0.5 m and a multi-spectral resolution of 2 m, along with its capability to perform highly agile and multi-mode imaging. Its image products can be subscribed to for a fee and can present ground details [21], mainly used to investigate the different types of ground features in the study area, to establish the locations of rice field and water edge samples, and to verify the retrieval accuracy.

Finally, the influence of altitude on the rice harvesting period was investigated with the digital elevation data and rice harvesting period data. The digital elevation data are taken from the NASA website [22] at a spatial resolution of 12.5 m. We determined the specific rice harvesting period in different regions by employing a combination of information search and on-site investigation methods, including consulting relevant news reports and government announcements and conducting interviews with residents.

### 2.3. Methodology

#### 2.3.1. Analysis of Rice Field Data Extraction Methods

Rice field data extraction methods can be divided into integrated extraction and individual extraction. The integrated extraction can be used with all land use types, but the resolution, data sources, classification criteria, and timing vary among different land use data products. Global land use data products include ESRI Land Cover data, GlobeLand30, CGLS-LC100 and MCD12Q1. Most of the classification methods are based on machine learning, with spatial resolutions ranging from 10 m to 500 m. GlobeLand30, ESRI Land Cover data originates from Sentinel-2, and its 10 m resolution is advantageous; however, the classification of some land classes is not sufficiently detailed, and there is a lack of data before 2017. GlobeLand30 is an important global land cover dataset at a 30 m resolution, and its data sources include TM5, ETM+ and OLI [23]. However, the product currently only includes data from 2000, 2010 and 2020. The CGLS-LC100 and MCD12Q1 products are more commonly used. Their data sources are PROBA-V and MODIS, respectively. However, their resolution is lower than ESRI Land Cover data and GlobeLand30. Considering the aim of rice field data extraction, all four of the above global land use data products only identify farmland or arable land without further subdivision to rice fields. The spatial resolution of the National Land Survey data is better than 1 m, and the classification criteria are more comprehensive and detailed. Cultivated land is classified into rice fields, irrigated land and dry land [24]. The rice field subdivisions include rice fields under cultivation and abandoned rice fields. The data extraction method is mainly by manual visual interpretation, and its classification accuracy is high. There are some disadvantages, such as long processing duration, high cost and slow data update.

The analysis of the available data products shows that it is difficult to extract rice field data directly based on land use data. Even the Third National Land Survey, which has more comprehensive and detailed classification criteria, can only classify up to categories that include rice fields under cultivation and abandoned rice fields. The Third National Land Survey utilizes manual visual interpretation to perform the classification, resulting in high classification accuracy. However, this method is costly and time-consuming, making it more suitable for small-scale land use classification. Most of the data products are classified by machine learning but are mainly applied to cultivated land, so the individual extraction of rice fields is more usually combined with the characteristics of the rice fields.

The characteristics of the terrain environment, the complexity of land use types and the degree of fragmentation in different study areas necessitate machine learning combined with the phenological characteristics of rice fields to extract rice information [25,26]. However, the extraction accuracy, constraints, and difficulty of different methods vary for different projects and are heavily influenced by the specific study area. The phenological characteristics of rice fields can be better represented in the data with short intervals and continuous time. Therefore, factors such as time resolution and the revisiting period of the satellite need to be taken into account when selecting the data source. Sentinel-2 provides the valuable advantage of offering free global remote-sensing data with high spatial resolution, good spectral quality and high time resolution, as it carries the Multispectral Imager (MSI) and is used for land and coastal observation [27]. At the same time, the growing season of rice is largely affected by cloud cover, which poses a challenge for optical sensors to capture cloud-free images [28]. But the Sentinel-2 satellite’s high revisit rate ensures that cloud-free observations of a given surface can be obtained at least once a month, barring the region and period with the highest cloud cover [29]. Additionally, Sentinel-2 imagery encompasses data captured from three bands that fall under the red-edge range. This unique feature allows for the provision of highly sensitive spectral information in vegetation monitoring studies, leading to a noteworthy enhancement in the precision of vegetation classification [30]. Therefore, it is very advantageous to use Sentinel-2 data as the basis for analyzing the phenological characteristics of rice fields.

#### 2.3.2. Analysis of the Intra-Annual Variation Characteristics of NDVI for Different Types of Ground Features

NDVI can detect the basic conditions of vegetation, including vegetation growth status and vegetation cover, and can be constructed as a time series curve to show the changes in vegetation in time and space [31,32,33,34,35]. The phenological characteristics and related information can be extracted. The study area was divided into five ground feature types based on the remote-sensing high-resolution images. They were rice fields, dry land, water bodies, construction land and forest and grassland (Table 1).

**Figure 2:**
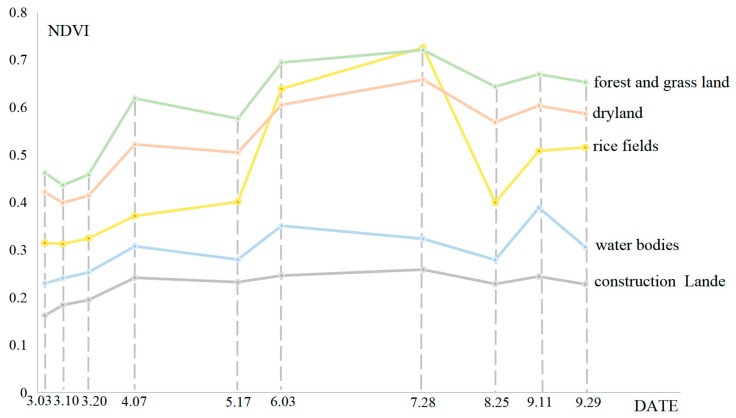
The time-series NDVI curves of various types of ground features in the study area.

The NDVI values are calculated as in Equation (1) [36], where NIR is the near-infrared band and R is the red band. In Sentinel-2, NIR and R are B8 and B4 bands, respectively. The NDVI values of various types of ground features were obtained by manual visual interpretation, and the time-series NDVI curves were constructed (Figure 2). The value and its variation were influenced by weather, such as persistent high temperatures and rainy days.

(1) $$\mathrm{NDVI}=\frac{NIR-R}{NIR+R}$$

**Figure 3:**
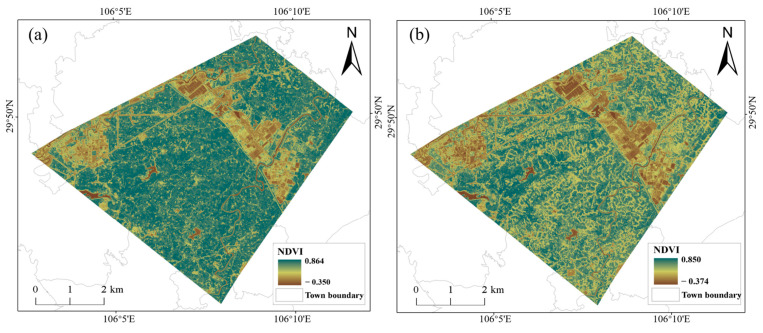
(**a**) NDVI images in the study area before the rice harvesting period. (**b**) NDVI images in the study area after the rice harvesting period.

The NDVI values of forest and grassland in the study area are generally higher than those of other land types, while the NDVI values of rice fields and dryland are in the middle, and the NDVI values of construction land and water bodies are the lowest (Figure 2). From the analysis of the trend and degree of change, the NDVI values of rice fields decreased significantly between 28 July 2019 and 25 August 2019. After data collection and field investigation, it is found that the rice is sown in early April, transplanted in late April and early May, enters the nodulation and gestation period in June, and reaches the spike stage in July. The spike stage marks the shift from root, stem and leaf growth to flowering and fruiting [37]. The rice begins to mature for harvest in August. The period when the NDVI values of the rice fields decrease significantly is around the time of harvesting. Then, the NDVI images are compared in the study area between pre-harvest and post-harvest periods of rice fields (Figure 3), Figure 3a is on 28 July 2019 and Figure 3b is on 25 August 2019. It is found that rice fields grow substantially, and the NDVI values are higher near the harvesting period, and after the harvesting period, the NDVI values are lower. During this period, the NDVI values of other types of ground features change only slightly. Therefore, rice fields were extracted based on the difference in NDVI between the pre-harvest and post-harvest periods. There were two problems: firstly, to determine the sentinel data between the pre-harvest and post-harvest periods of rice fields to be used, and secondly, to determine the threshold value of NDVI difference between the pre-harvest and post-harvest periods.

#### 2.3.3. Spatial and Temporal Simulation of the Rice Harvesting Period

**Figure 4:**
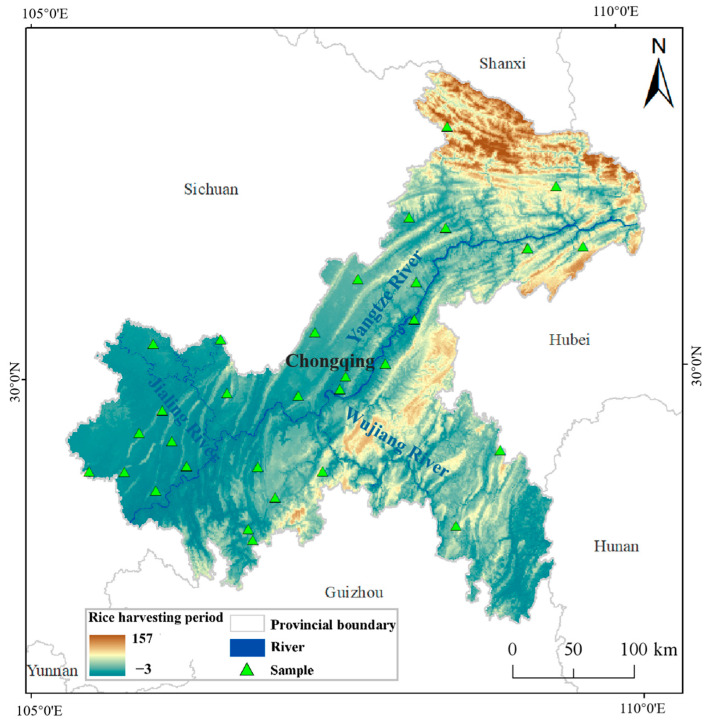
Numerical simulation of the rice harvesting period in Chongqing.

To determine the sentinel data to be used between the pre-harvest and post-harvest periods, it was first necessary to specify the rice harvesting period. From research and field surveying, it was found that the altitude and latitude of the region influence the rice harvesting period, so a model of the rice harvesting period was constructed using two indicators of altitude and latitude [38,39]. The rice harvesting period in each region was a time range, but since the subsequent study would use a multiple regression analysis, a value between the pre-harvest and post-harvest period of rice fields was temporarily used to represent the harvesting period. A total of 32 sample points were randomly selected, and the relevant information was collected to determine the altitude, latitude and harvesting period of the sample points (Figure 4). Because the rice harvesting period was affected by two variables (altitude and latitude), partial correlation analyses were performed using SPSS for the three indicators of altitude, latitude and harvesting period. Partial correlation analysis means that when exploring the correlation between one indicator and the rice harvesting period, another indicator is used as a control variable, thus eliminating its influence on the correlation and analyzing only the degree of correlation between the independent variable and the harvesting period [40,41].

The correlation analysis showed that the correlation between altitude and harvesting period was 0.978 when latitude was used as the control variable. The correlation between latitude and harvesting period was 0.922 when the altitude was used as the control variable. Both of the correlations were significant at *p* < 0.001, indicating that altitude and latitude were significantly correlated with the harvesting period. Multiple linear regression analysis was used to construct a mathematical model of the rice harvesting period and the two indicators. The independent variables (altitude and latitude) explained 96% of the dependent variable (harvesting period). This result indicated that the fitting effect was good. In the analysis of variance, $p<0.001^{b}$, b is the predictor variable, showed that the fitted equation was statistically significant. The significance of the constant, latitude and altitude coefficients were less than 0.001, indicating that the regression effect of the equation was significant. Finally, the relationship between the rice harvesting period and altitude and latitude was derived as:

(2) $$y=8.077\times X_{1}+0.056\times X_{2}-245.040$$

where $X_{1}$ is the latitude, $X_{2}$ is the altitude, and Y is a value between the pre-harvest and post-harvest period of the rice fields. When the altitude and latitude of a certain location are substituted into Equation (2) and Y = 1 is obtained, it means that the rice was harvested around 1 August. A numerical simulation of the rice harvesting period in Chongqing was constructed according to Equation (2). The rice harvesting period in the western part of Chongqing is mainly in early to mid-August, with some harvesting in mid to late August (Figure 4). The rice harvesting period in the middle of the northeast and southeast directions is mainly in late August and early September. It is important to point out that even though Figure 4 covers the entirety of Chongqing, not all areas are suitable for rice cultivation. These unsuitable parts are still assigned a rice harvesting period in Figure 4 but do not have practical relevance.

The value obtained according to Equation (2) was between the pre-harvest and post-harvest periods, so the number of days needed to be added or subtracted before and after this value. The resulting range could be used as the harvesting period for the study area. Since the normal distribution is within plus or minus three standard deviations (99.6%), the value of three standard deviations can be added or subtracted to estimate the rice harvesting period [42]. The standard deviation in the multiple regression was the error of the standard estimation of 2.749. The final model of the rice harvesting period was obtained:

(3) $$y=8.077\times X_{1}+0.056\times X_{2}-245.040\pm 8.247$$

The aim of adding or subtracting three standard estimates was to reduce the error, and the resulting range was usually larger than the actual harvesting period. In real farming (as opposed to modeled), extreme weather, such as continuous high temperatures or rain, can impact the timing of the rice harvesting period [43]. By also considering the average elevation of the study area of 242.81 m and latitude of 29.82° in the model, the harvesting period in the area was calculated to be between 1 August and 18 August. This harvesting period set a baseline for determining the Sentinel data between the pre-harvest and post-harvest periods to be used in the study area. Taking into account the harvesting period, the quality of the data, and the difficulty of acquisition, this paper used the Sentinel-2 L1C product with a resolution of 10 m from 28 July 2019 and 25 August 2019 as the data source.

#### 2.3.4. Rice Field Data Extraction Model Construction and Application

**Figure 5:**
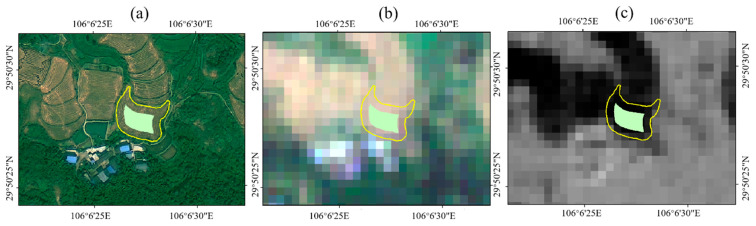
(**a**) The boundary and inside of the sample region of the rice field are displayed on the SuperView-1 image taken on 25 August 2019. (**b**) The boundary and inside of the sample region of the rice field are displayed on the Sentinel-2 image taken on 25 August 2019. (**c**) The boundary and inside of the sample region of the rice field on the NDVI difference image between the preharvest and postharvest period of rice fields.

Based on the unique features of large differences in vegetation between the pre-harvest and post-harvest periods of rice fields, the extraction model of rice fields was constructed (Equation (4)):

(4) $${Diff}_{NDVI}={NDVI}_{t_{1}}-{NDVI}_{t_{2}}$$

where ${NDVI}_{t_{1}}$ is the NDVI values of the preharvest period of rice fields, ${NDVI}_{t_{2}}$ is the NDVI value of the postharvest period of rice fields. To determine the threshold value of ${Diff}_{ndvi}$ for extracting the initial extent of rice fields, we can use “a” to represent the threshold value. If greater than “a”, these ground features are rice fields, or conversely, the features are other land types. The Sentinel data between the pre-harvest and post-harvest periods of rice fields were determined based on the rice harvesting period, 28 July to 25 August 2019. Based on the extraction model of rice field data, calculated the value of $Diff_{NDVI}$. To determine the threshold value of rice fields, 33 samples of the rice fields were randomly selected from the high-resolution images in the study area, and the sample regions of $Diff_{NDVI}$ were statistical by the partition statistics tool in ArcMap. Because the sample regions’ boundaries were mixed pixels, the boundaries and insides of the region of $Diff_{NDVI}$ needed to be analyzed independently, and obtained the threshold as accurately as possible. Buffer zones of 10 m (the pixel size of the ${Diff}_{NDVI}$) inside the boundaries were made, and the relative areas of the buffer zones were extracted based on the rice field samples as the total areas (Figure 5). Figure 5 shows the difference of the same sample in different periods, the yellow line is the boundary of the sample area, and the green area is the interior of the sample area. Figure 5a shows a sample of the SuperView-1 image taken on 25 August 2019, Figure 5b shows a sample of the Sentinel-2 image taken on the same date, and Figure 5c shows a sample of the NDVI difference between the preharvest and postharvest period of rice fields.

The average and standard deviation of the boundaries and insides of the sample regions of the rice fields of $Diff_{NDVI}$ were calculated separately and three standard deviations were subtracted from the mean to obtain a boundary threshold of 0.197 and an inside threshold of 0.317. The threshold of rice fields of $Diff_{NDVI}$ was 0.197, and this was used to extract the rice fields.

#### 2.3.5. The “Water Edge Effect” and Its Treatment

**Figure 6:**
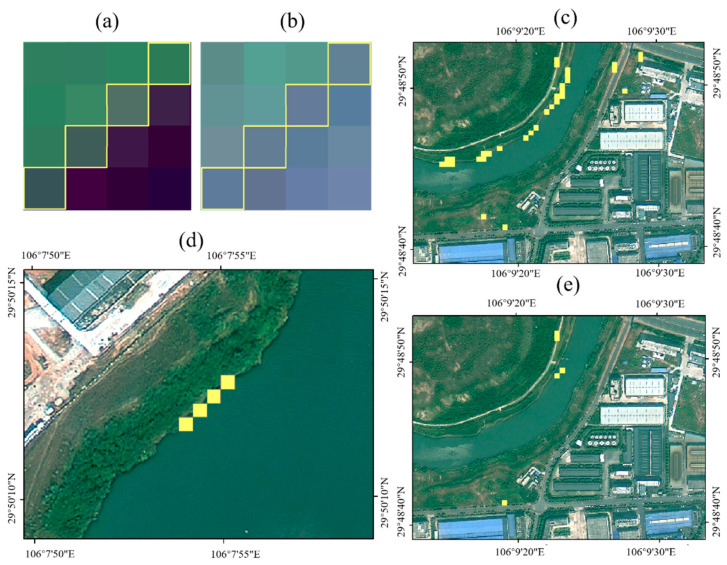
(**a**) The pixels that experience the “water edge effect” are displayed on the Sentinel-2 image taken on 28 July 2019. (**b**) The pixels that experience the “water edge effect” are displayed on the Sentinel-2 image taken on 25 August 2019. (**c**) The image before the removal of the “water edge effect” pixels. (**d**) The pixels that experience the “water edge effect” are displayed on the SuperView-1 image taken on 25 August 2019. (**e**) The image after the removal of the “water edge effect” pixels.

Rice fields extracted only by the difference of NDVI value between the pre-harvest and post-harvest periods show the “water edge effect”, whereby a large amount of the water edge is extracted along with the rice fields (Figure 6). The yellow pixels (yellow lines and yellow squares) in Figure 6 are hybrid pixels demonstrating the water edge effect. Figure 6d shows the position of these pixels on 25 August 2019 for the SuperView-1 data. Figure 6a,b show the position of these pixels on 28 July and 25 August 2019, respectively, for the Sentinel-2 data. The mixed pixels with the water edge effect were closer to the onshore pixels on 28 July 2019, and these mixed pixels were closer to the water body pixels on 25 August 2019. The SuperView-1 satellite data show that these pixels were closer to the water body on 25 August 2019. This finding indicates that from the pre-harvest to the post-harvest period of rice, the mixed pixels with the water edge effect change from shore pixels to water body pixels, and most of the shore pixels of the type ground feature are dry land or forest and grassland. The difference in the NDVI between the dryland pixels, forest and grassland pixels, or water body pixels, and the distinct change in NDVI of rice from preharvest to postharvest yields the “water edge effect”. The change of the types of ground features of mixed pixels is mainly due to the change of temperature and precipitation between the pre-harvest and post-harvest periods of the rice fields, which causes the boundary of the water bodies to shift.

The water edges were extracted by calculating the NDWI values according to Equation (5) [44,45]. The calculation of the NDWI values is a process of normalized difference processing with specific bands from the remote sensing image to highlight the water bodies in the remote sensing image [46]. Construction lands interfered with the water bodies when we applied the NDWI values to extract the water bodies. However, there were almost no construction lands in the initial rice fields data, so removing the water edges extracted by the NDWI values from the initial rice fields data, the mis-scored construction lands did not affect the results.

(5) $$\mathrm{NDWI}=\frac{\mathrm{Green}-NIR}{Green+NIR}$$

where NDWI value is the value of the green waveband minus the NIR waveband divided by the green waveband plus the NIR waveband, green is the green waveband and NIR is the near-infrared waveband.

The NDWI values are calculated using 28 July 2019 Sentinel-2 data. A total of 20 samples of water edges were randomly selected by high-resolution remote sensing images of the same period, and the NDWI values of the water edge sample regions were statistical. The pixels of the water edge effect was mainly the mixed pixels generated by the junction of water bodies and shore. To aid statistical analysis, widened the water edges, and buffer zones of 2 m were made with the boundaries of samples of water edges. The average and standard deviation of the NDWI values of buffer zones were calculated separately, and three standard deviations were added and subtracted from the average. The NDWI range of the boundaries of water bodies was (−0.533, −0.207). Finally, the pixels of water edges were removed from the initial data of rice fields, totaling 11,051 and accounting for 8.52% of the initial data. Figure 6c shows the effect before the removal of water edges, and Figure 6e shows the effect after the removal of water edges. There is a small part of the water edge that cannot be erased.

## 3. Results

### 3.1. Precision Evaluation

**Figure 7:**
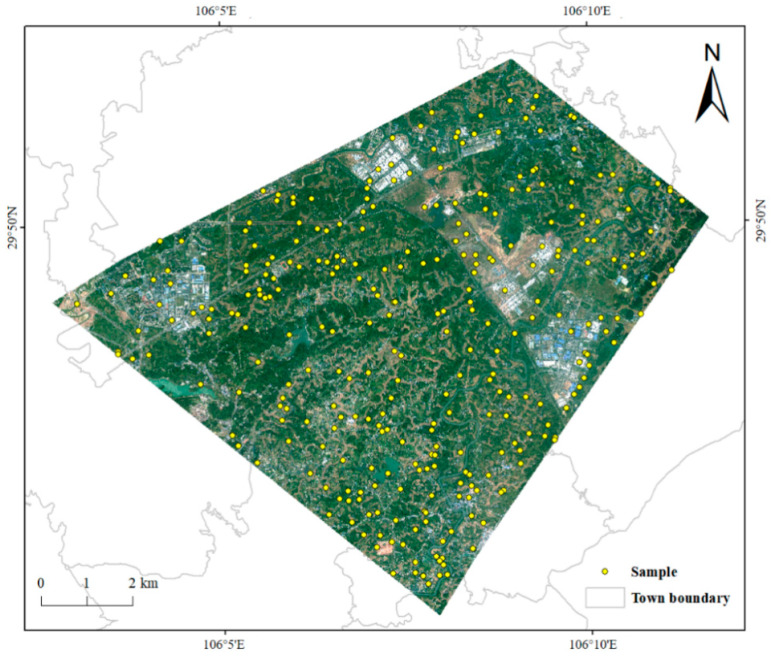
Visual interpretation of the distribution of sample points.

In this paper, we primarily use the tool “Select the Ground Truth Classification lmage” in ENVI for stratified sampling and randomly generate 300 validation points, including 100 for the rice fields layer and 200 for the non-rice fields layer (Figure 7). Using SuperView-1 with a resolution of 0.5 m, the verification points are visually interpreted to obtain the classification accuracy as well as the Kappa coefficient by using the “Calculate Confusion Matrix” tool in the “Image Segmentation and Classification” toolset in ArcMap.

From the 88 verification points of the manual visual interpretation of rice fields, 86 have correctly classified ground feature types and 2 are misclassified to other land types, giving a classification accuracy of 97.73%. From the 212 verification points of the manual visual interpretation of other land types, 198 points are correctly classified and 14 are misclassified as rice fields. The final classification accuracy is 93.40%, the overall accuracy is 94.67%, and the Kappa coefficient is 0.88 (Table 2).

### 3.2. Results of Rice Fields Extraction

**Figure 8:**
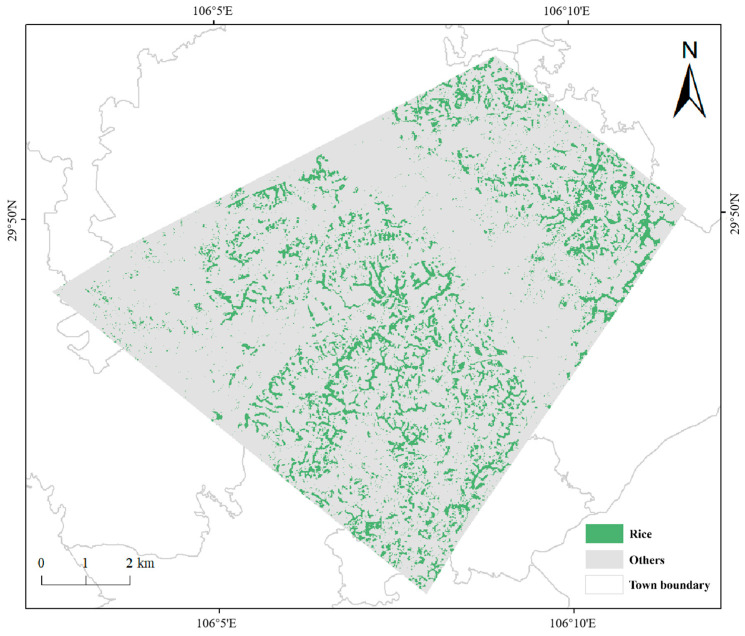
Distribution of rice fields in the study area in 2019.

Through the analysis of the time-series NDVI curves of various types of ground features in the study area, it is found that the NDVI values of various types of ground features are highly variable and characterized by a decreasing distribution. The NDVI values of rice fields decrease significantly, with a difference of 0.33, the other types of ground features are affected by the summer drought, and NDVI values also show a decreasing trend, but the change is small, with a difference of 0.09 for drylands, 0.04 for water bodies, 0.03 for construction land and 0.08 for forest and grassland. This result indicates that the degree of variation of NDVI values in rice fields during this period differs from other land types. For the rest of the period, the trends of NDVI values and the degrees of change for all types of ground features are similar. Because rice fields grow substantially and the NDVI values are higher near the harvesting period, and after the harvesting period, the NDVI values are lower. During this period, the NDVI values of other types of ground features change only slightly. Lastly, rice fields with an area of 11.86 km^2^ are identified based on this characteristic, accounting for 15.82% of the total study area (Figure 8). With the exception of large patches of construction lands in the northeast and west of the study area, rice fields are evenly distributed in the remaining regions. Rice fields are more contiguous, but there are some scattered rice fields that are planted sporadically. The accuracy of rice field extraction is 94.67%, indicating that the NDVI difference method based on sentinel data for rice field extraction is excellent. However, 14 of 212 points are misclassified as rice fields, indicating that a small number of the other types of ground features still existed in the extracted rice fields.

## 4. Discussion

### 4.1. Effect of External Factors on the Extraction Results of Rice Fields

NDVI variation forms the core of the difference method. However, certain external factors that cannot be entirely eliminated will inevitably interfere with the NDVI variation. These factors may include extreme weather conditions, human activities, and others that may have a significant impact on the NDVI variation [47]. Some of these external factors affect NDVI values by advancing or delaying the rice growth process. Examples of such factors include continuous high temperatures or persistent rain. On the other hand, certain factors can directly interfere with NDVI values, including changes in groundwater level and soil moisture caused by irrigation, water pumping, or other natural causes. These factors can significantly affect NDVI values. To mitigate the impacts of external factors on the early or delayed rice harvesting period, the value of three standard deviations can be added or subtracted in the harvesting period model to make the rice harvesting period conform to reality. However, this paper does not take a more feasible approach to deal with the factors that directly interfere with the NDVI values. At the same time, there are few studies on the relationship between NDVI and soil and water environmental factors in agricultural planting areas [48], which will be improved in subsequent studies.

### 4.2. Effect of the Rice Harvesting Period on the Extraction Results of Rice Fields

The NDVI difference method proves to be more convenient when applied in areas with similar rice harvesting periods. In the study, the difference between the pre-harvest and post-harvest periods was determined to be within five days; thus, rice fields can be harvested in a single operation. On the contrary, there are large differences in rice harvesting periods in one study area, which can quickly clarify the rice harvesting period of each location through the harvesting period model. We then combine the places with similar harvesting periods as a region, and then rice fields are extracted separately from different regions.

### 4.3. Effect of Mixed Pixels on the Extraction Results of Rice Fields

Between the pre-harvest and post-harvest periods, because of factors such as temperature and precipitation changes, the boundary of the water bodies move, resulting in changes in the NDVI values of some of the water edge pixels. The changed parts overlap with the different range of the NDVI values between the pre-harvest and post-harvest periods of rice fields, thus creating the “water edge effect”. NDWI method can effectively eliminate the water edge effect, improving the accuracy of the rice field extraction. The pixels of the “water edge effect” are, however, not completely eliminated. Other types of ground feature boundaries still have mixed pixels that affect the extraction accuracy of rice fields. Yang et al. [49] used the NDWI and NDVI together to obtain information about rice fields in Laian County, Anhui Province, and the extraction accuracy was 92.79%. This method is based on the characteristics of rice fields with high water content in the lower bedding surface during early growth and the sparse to lush rice from the tillering stage to the tasseling stage. As same as this paper, this model only used only specific phenological periods and a small number of indicators to study the distribution of rice, and it has good generality; however, the types of ground feature boundaries also tend to create mixed pixels, which affects the accuracy of the rice field data. Singha M et al. [50] evaluated the utility of time features extracted from coarse-resolution data in the fine-resolution object-based classification of rice fields in five selected regions of northeast India. The overall classification accuracy was 84.37%, and the kappa coefficient was 0.68. The results indicated the importance of feature selection for achieving higher classification accuracy and the potential for accurate rice classification through the combined use of time and spectral features. This article achieved good extraction results for rice by combining the features of time and NDVI. However, mixed pixels were not completely removed in this study, which had an impact on accuracy. Future research should investigate the problem of mixed pixels.

## 5. Conclusions

This study has extracted the rice fields in the west of Chongqing using the NDVI difference method based on Sentinel data and reached the following conclusions:

1. The rice harvesting period is significantly correlated with altitude and latitude. A simulation model of the rice harvesting period is constructed by multiple regression analysis that can effectively determine the best period of remote sensing images needed to extract rice fields.
2. The confusion matrix shows that the overall accuracy is 94.67% and the Kappa coefficient is 0.88, indicating that this method has a better extraction effect.
3. The mixed pixels have a large impact on the accuracy of rice field extraction due to the “water edge effect”. NDWI method can effectively eliminate the water edge effect, improving the accuracy of the rice field extraction.

## Figures and Tables

**Table 1:** Remote sensing image interpretation types.

Types of Ground Features	Actual Inclusion Types
Rice fields	Rice
Dry land	Crops other than rice
Water bodies	Rivers, lakes, reservoirs, ponds and ditches
Construction land	Bare ground, buildings and roads
Forest and grass land	Grasslands, shrubs and woodlands

**Table 2:** Confusion matrix of classification result of rice fields in the study area based on SuperView-1 data image.

ClassValue	C_others	C_rice	Total	U_Accuracy	Kappa
C_others	198	2	200	0.99	/
C_rice	14	86	100	0.86	/
Total	212	88	300	/	/
P_Accuracy	0.93	0.98	/	0.95	/
Kappa	/	/	/	/	0.88

## Data Availability

The Sentinel-2 data that support the findings of this study are openly available in USGS at https://earthexplorer.usgs.gov/ (accessed on 12 April 2022). SuperView-1 is a domestically developed 0.5-m commercial remote sensing satellite in China. Its image products are available for a paid subscription and can be ordered through the website https://www.gscloud.cn/ (accessed on 12 April 2022). The digital elevation data that support the findings of this study are openly available in NASA at https://search.asf.alaska.edu/ (accessed on 12 April 2022). The data about the rice harvesting period in the study area are obtained through an information search and field survey.
